# Leveraging transcriptomics for precision diagnosis: Lessons learned from cancer and sepsis

**DOI:** 10.3389/fgene.2023.1100352

**Published:** 2023-03-10

**Authors:** Maria Tsakiroglou, Anthony Evans, Munir Pirmohamed

**Affiliations:** ^1^ Department of Pharmacology and Therapeutics, Institute of Systems, Molecular and Integrative Biology, University of Liverpool, Liverpool, United Kingdom; ^2^ Computational Biology Facility, Institute of Systems, Molecular and Integrative Biology, University of Liverpool, Liverpool, United Kingdom

**Keywords:** biomarker, cancer, diagnosis, sepsis, transcriptomics

## Abstract

Diagnostics require precision and predictive ability to be clinically useful. Integration of multi-omic with clinical data is crucial to our understanding of disease pathogenesis and diagnosis. However, interpretation of overwhelming amounts of information at the individual level requires sophisticated computational tools for extraction of clinically meaningful outputs. Moreover, evolution of technical and analytical methods often outpaces standardisation strategies. RNA is the most dynamic component of all -omics technologies carrying an abundance of regulatory information that is least harnessed for use in clinical diagnostics. Gene expression-based tests capture genetic and non-genetic heterogeneity and have been implemented in certain diseases. For example patients with early breast cancer are spared toxic unnecessary treatments with scores based on the expression of a set of genes (e.g., Oncotype DX). The ability of transcriptomics to portray the transcriptional status at a moment in time has also been used in diagnosis of dynamic diseases such as sepsis. Gene expression profiles identify endotypes in sepsis patients with prognostic value and a potential to discriminate between viral and bacterial infection. The application of transcriptomics for patient stratification in clinical environments and clinical trials thus holds promise. In this review, we discuss the current clinical application in the fields of cancer and infection. We use these paradigms to highlight the impediments in identifying useful diagnostic and prognostic biomarkers and propose approaches to overcome them and aid efforts towards clinical implementation.

## 1 Introduction

Precision diagnosis recognises the individuality among patients in their clinical pathway by the simultaneous analysis of multimodal data with artificial intelligence (Kline et al., 2022). Precision molecular diagnostics also guide efficient, safe and cost-effective therapeutics (Ho et al., 2020). Oncology has been at the epicenter of these developments (Wahida et al., 2023), while precision approaches in infectious diseases at the research and clinical level may help in tackling an imminent antibiotic crisis (Cook and Wright, 2022). The importance of molecular technologies has been underlined in the severe acute respiratory syndrome coronavirus 2 (SARS-CoV-2) pandemic (Berber et al., 2021; Wang et al., 2021), but it also highlighted the need to increase our diagnostic capacity (McDermott et al., 2021).

There is an unprecedented abundance of heterogenous data available at the clinical (electronic health records) and molecular (-omic databases) level, but occasionally phenotypic information is incomplete to assist interpretation of high through-put data (Haendel et al., 2018). The interrogation of DNA has been under investigation as a diagnostic modality for a few decades with increasing translation into clinical care (Pirmohamed, 2023) and measurement of protein products is common practice. For instance, a combination of gene markers and a panel of proteins in CancerSEEK (Cohen et al., 2018) and methylation of circulating tumour DNA (Jin et al., 2021) are breakthroughs in early detection of solid tumours and colorectal cancer, respectively. However, other molecular modalities of genetic information, such as RNA, have been explored to a lesser extent for clinical application.

In the era of precision medicine, misdiagnosis is still common in clinical practice. In a US national epidemiologic study, serious diagnostic errors resulting in significant harm were higher for certain conditions such as spinal abscess, aortic aneurysm and dissection and lung cancer (rate per incident case of disease: 36%, 17%, and 14%, respectively) (Newman-Toker et al., 2021). A gold standard test is widely accepted as the best available method to determine the presence of a condition, but it often lacks true 100% accuracy and it succumbs to advances in knowledge and technology (Sox et al., 2013; Porta, 2016). A “good” diagnostic test should also be scalable, cost-effective, and timely. There is no doubt that we need improved diagnostic tools to guide personalised management of patients and new technologies hold promise towards that direction (Love-Koh et al., 2018). But new advances also lead to many challenges. For instance, systems science, where coupling of the molecular world with mathematics, allows the modelling of multiple components and their interactions, has the potential to replace traditional reductionist approaches focusing on a single molecule (Hasin et al., 2017). However, such technologies generate vast amounts of raw large-scale data, which is incomprehensible if not analysed, integrated and interpreted with advanced bioinformatic methods and computational tools (Apweiler et al., 2018). Such approaches may not only lead to more precise diagnosis but will also generate accessory information that may enable better understanding of mechanistic pathways, disease processes, new biomarkers and druggable targets. The major challenge apart from interpretation is how such technologies can be implemented into clinical care (Green et al., 2020). But fortunately, there are some sentinel areas where novel diagnostics have been introduced (Cohen et al., 2018; Buus et al., 2021), and we need to learn lessons from the implementation process to enable uptake of novel diagnostics in other disease areas in the future.

This narrative review attempts to summarise the potential benefits and challenges of implementation of transcriptomic-based technologies into clinical settings. Cancer (in particular breast cancer) and sepsis are the two areas where gene expression tests have been developed from bulk RNA exploration. We use these paradigms to highlight the impediments in identifying useful diagnostic and prognostic biomarkers and propose ways to circumvent difficulties in the translational pathway.

## 2 The transcriptome

### 2.1 The basics of the transcriptome

The transcriptome is the total set of expressed RNA in a cell or population of cells at a specific time point. Mature messenger RNA (mRNA), which is the interim carrier of information between the genome and protein, is transcribed from a very small fraction (less than 2% to 3%) of cellular DNA (International Human Genome Sequencing Consortium, 2004). Multiple regulators decide the fate and character of the message passed on to form proteins through various mechanisms including alternative splicing and RNA editing (de Hoon et al., 2015; Abascal et al., 2020). The regulation of the whole machinery is extremely complex and involves long non-coding RNAs (lncRNAs), microRNAs (miRNAs), transfer RNAs (tRNAs), ribosomal RNAs (rRNAs), small nuclear RNAs (snRNAs), small nucleolar RNAs (snoRNAs), short interfering RNAs (siRNAs) and other transcripts. Furthermore, high throughput technologies have identified a plethora of novel RNA molecules but their involvement in various cellular activities is unclear (Pertea, 2012; Palazzo and Koonin, 2020).

RNA is the most dynamic cellular component regulating gene expression through complex processes including transcription, maturation and degradation (Cao and Grima, 2020). Transcription mostly occurs intermittently (on/off promoter switches) and the size and frequency of transcription bursts contribute to the molecular phenotype of a cell at a particular time point (Eling et al., 2019). Deterministic factors drive the mean expression of a gene without accounting for stochastic processes (Kærn et al., 2005). However, intrinsic molecular fluctuations (stochastic noise) have been linked to important processes such as cell fate, immune plasticity, ageing and cancer development. The combination of deterministic and stochastic components drives non-genetic heterogeneity which is modulated by gene-regulatory circuits and results in variability in transcript abundance across seemingly homogenous cell populations (Eling et al., 2019). Although there is an inverse correlation between mean gene expression and fluctuation, it has been recently shown that changes in transcriptional noise can initiate cell re-programming and development while mean gene expression remains stable (Desai et al., 2021). Collectively, therefore, RNA corresponds to a snapshot of the cellular state and has enormous potential for application to clinical diagnostics (Byron et al., 2016).

### 2.2 Technologies measuring the transcriptome

Technological advancements have enhanced our understanding of the transcriptome. Reverse transcriptase polymerase chain reaction (RT-PCR) is considered a gold standard for detecting qualitatively and quantitatively a limited number of transcripts (Dramé et al., 2020). Microarrays have revolutionised our approach to RNA measurement by using probes on a solid surface that hybridise with thousands of transcripts (Schena et al., 1995). They were recognised as a key tool in advancing personalised medicine (Shi et al., 2006), but despite 25+ years of development, their clinical utility remains limited (Piccart et al., 2021). Variation in sample preparation decreases reproducibility and background noise obscures the detection of low signal transcript expression. RNA sequencing (RNA-seq) technologies are more powerful tools as pre-defining RNA targets is not required and they have a greater dynamic range. RNA-seq allows the detection of the diversity in the transcriptome through the quantification of known and novel transcripts regardless of their abundance, including non-coding RNA, single nucleotide variants, fusion genes and splice variants (Byron et al., 2016). Moreover, RNA-seq at the level of single cell (scRNA-seq) allows the detection of previously unexplored processes such as transcriptional noise (Eling et al., 2019; Desai et al., 2021). Although RNA-seq outperforms microarrays in assessing complex gene expression profiles, prediction of clinical endpoints is not affected by the platform (Zhang et al., 2015), and data based on microarray experiments have driven a plethora of discoveries. A caveat of whole RNA sequencing is its relatively poor ability to identify and quantify low abundance transcripts. Probe-based assays targeting genes of interest, such as RNA CaptureSeq have been developed to fill this gap along with sophisticated bioinformatic algorithms aiming to increase detectability of unknown sequences (Grioni et al., 2019).

Gene expression profiling provides an enormous amount of high-resolution data from a single experiment. The size of the human transcriptome remains debatable with the majority of it referred to as “dark matter” because its function is unknown (Kapranov and St Laurent, 2012). RNA-seq exceeds the size of the human genome by generating up to six billion short reads and their assembly into the transcriptome is a challenging task (Pertea, 2012). Complex computational algorithms are deployed at multiple stages of data analysis and require bioinformatics expertise (Kukurba and Montgomery, 2015). The analytical pipelines attempt to identify a set of informative genes to guide the elucidation of novel molecular mechanisms, the development of prognostic and predictive biomarkers and the identification of druggable targets.

### 2.3 Clinical utility of the transcriptome

Over 100 genetic tests for 30 conditions in the field of oncology, haematology, genetic disorders and pharmacogenetics, have received FDA approval to date. Less than ten tests are based on RNA measurement and only four utilise gene expression profiles with more than two RNA targets (FDA, 2021).

Molecular diagnostics focusing on the genome suffer from a limited ability to reflect accurately the *in vivo* variability within a condition at a particular time-point and among patients. The hallmark of acute lymphoblastic leukaemia (ALL), for instance, is numerous genetic aberrations stratifying patients into prognostic and therapeutic groups (Pui et al., 2019). However, multiple mutations identified at the genome level may not be contributing to the disease. Transcriptome sequencing characterises clinically relevant genomic alterations and variants in real time with higher sensitivity compared to whole-genome sequencing and it has been crucial in the discovery of novel subtypes and therapy tailoring (Roberts and Mullighan, 2015). Gene expression studies identified the Philadelphia chromosome-like ALL subtype and the downstream involvement of kinases guiding the use of tyrosine kinase inhibitors (TKI) (Inaba et al., 2017).

Transcriptomics is explored as a complementary method to genomic testing for precision-based treatments in cancer patients (Lee et al., 2021; Tsimberidou et al., 2022). The Worldwide Innovative Network (WIN) study to select rational therapeutics based on the analysis of matched tumour and normal biopsies in subjects with advanced malignancies (WINTHER, NCT01856296) was the first large-scale prospective clinical trial that allowed a fraction of patients with no actionable DNA alterations to have RNA-guided treatments using a novel algorithm (Rodon et al., 2019). The Individualised Therapy For Relapsed Malignancies in Childhood (INFORM) registry collects real-world clinical and multi-omic data from routine biopsies to translate them to precision treatments and inform future clinical trials (van Tilburg et al., 2021). The first trial (NCT03838042) is ongoing and investigates the combination of Nivolumab and Entinostat in children and adolescents with refractory high-risk malignancies. Stratification of patients in accordance with their tumour genetic mutation and gene expression profiles will serve for the purposes of biomarker development and to minimise unnecessary risks in patients (van Tilburg et al., 2020).

Liquid biopsy of extracellular RNA (exRNA) has been embraced as a promising tool for screening and disease monitoring purposes and as an alternative to invasive methods of diagnosis such as tissue biopsy (Heitzer et al., 2019; Zhou et al., 2020; Wu et al., 2022). Although studies investigating exRNA in clinical application are scarce, recent developments in oncology are paving the way by enabling the distinction between tumour-specific RNA and total circulating extracellular transcriptome (Vermeirssen et al., 2022; Zong et al., 2023). Moreover, analysis of intracellular RNA of circulating tumour cells and peripheral blood mononuclear cells (PBMC) has identified prognostic pathways for response to treatment in patients with metastatic castration-resistant prostate cancer (Zhang et al., 2022).

## 3 Cancer

### 3.1 Transcriptomics in early breast cancer

In breast cancer, patient stratification based on expression of tumour markers (e.g., ER, PR and HER2 in breast cancer) has guided treatment strategies for over 30 years (Cardoso et al., 2016) laying the foundation for remarkable advances in molecular diagnostics (Buus et al., 2021). Early breast cancer (Supplementary Box S1) represents a successful paradigm of the applied knowledge accrued from transcriptomics in clinical practice. Only a small proportion of patients with oestrogen receptor positive (ER+) and lymph node negative (LN-) breast cancer benefit from adjuvant chemotherapy. Unfortunately, clinicopathological features poorly characterise ER+/LN- tumours and immunohistochemical techniques cannot be relied on to make treatment decisions (Fitzgibbons et al., 2000; Eifel et al., 2001). The standard practice has been to use a combination of hormonal and chemotherapy regimens, despite evidence suggesting that around 80% of patients were overtreated and unnecessarily exposed to chemotherapy and the potential toxicity (van 't Veer et al., 2002). Hence, identification of gene expression signatures able to predict risk of recurrence, and therefore stratify treatment, was a breakthrough in early breast cancer management (Schaafsma et al., 2021).

Commercially available assays, such as Oncotype DX (Genomic Health), MammaPrint (Agendia), EndoPredict (Myriad Genetics) and Prosigna (Nanostring Technologies) are endorsed by the UK National Institute for Health and Care Excellence (NICE) and international guidelines (Table 1). Expression levels of specific genes are measured in tumour samples with RT-PCR (Oncotype DX) or microarrays (MammaPrint) and a prognostic score is calculated with mathematical models in order to stratify patients into risk groups (Sparano et al., 2018; Piccart et al., 2021). EndoPredict produces a score based on both transcriptional and clinical (tumour size and nodal status) features. Prosigna classifies breast cancer into subtypes and calculates a score based on gene expression, subtype, clinical parameters (tumour size and nodal status) and proliferation pathways (Paik et al., 2006). Oncotype DX is based on a 21-gene signature which is independent of clinicopathological factors (Sparano et al., 2018). It is the only multi-gene assay which is validated to predict adjuvant chemotherapy benefit in addition to prognosis (Syed, 2020).

**TABLE 1:** Examples of commercialised gene expression tests and their characteristics.

Disease^a^	Trade name [manufacturer] (reference)	No of genes in signature	Platform	Clinical use	Guidelines^b^
Early breast cancer	Oncotype Dx (Genomic Health, now Exact Sciences) Paik et al. (2004), Sparano et al. (2018), Syed (2020)	21 (16 cancer-related and 5 reference genes)	RT-PCR	Prognostic of 10-year distant recurrence risk and predictive of adjuvant chemotherapy benefit in HR+/HER2-/LN ≤ 3	ASCO, ESMO [I, A], NCCN (Category 1) and NICE recommendation Cardoso et al. (2019), Henry et al. (2019), National Comprehensive Cancer Network (2021a), NICE (2018a)
	MammaPrint (Agentia) Cardoso et al. (2016), Piccart et al. (2021), van 't Veer et al. (2002)	70	Microarray	Prognostic of distant recurrence in women older than 50 years with HR+/HER2-/LN ≤ 3/T ≤ 5 cm	ASCO, ESMO [I, A] and NCCN (Category 1) recommendation (NICE does not recommend as it was not found to be cost-effective) Cardoso et al. (2019), Henry et al. (2019), National Comprehensive Cancer Network (2021a), NICE (2018a)
	Endopredict (Myriad Genetics)	12 (8 cancer-related and 3 reference genes)	RT-PCR	Prognostic of 10-year distant recurrence risk in HR+/HER2-/LN ≤ 3 treated with endocrine therapy alone	ESMO [I, B], NCCN (Category 2A) and NICE recommendation Cardoso et al. (2019), National Comprehensive Cancer Network, (2021a), NICE, (2018b)
	Prosigna (NanoString Technologies)	50 (+5 reference genes)	N-Counter^c^	Prognostic of 10-year distant recurrence in postmenopausal women with ER+/HER2-/LN ≤ 3.	ESMO [I, B], NCCN (Category 2A) and NICE recommendation Cardoso et al. (2019), National Comprehensive Cancer Network (2021a), NICE (2018b)
Prostate cancer	Oncotype DX (Exact Sciences)	17	RT-PCR	Prognostic of adverse pathology and 10-year risk of metastasis	ASCO, NCCN Eggener et al. (2020), National Comprehensive Cancer Network (2021c)
	Prolaris (Myriad Genetics; a combination of a gene expression score and a clinical score)	31 cell cycle progression genes (+15 control genes)	RT-PCR	Prognostic of 10-year risk of metastatic disease and prostate cancer-specific mortality	ASCO, NCCN and NICE advice MIB65 Eggener et al. (2020), National Comprehensive Cancer Network (2021c), NICE (2016)
	Decipher (Veracyte)	22	Microarray	Prognostic of adverse pathology, 10-year risk of metastasis and 15-year risk of prostate cancer-specific mortality	ASCO, NCCN Eggener et al. (2020), National Comprehensive Cancer Network (2021c)
Colon cancer	Oncotype DX (Exact Sciences)	12 (7 cancer-related and 5 reference genes)	RT-PCR	Prognostic of recurrence in stage II and III colon cancer	Not recommended National Comprehensive Cancer Network (2021b)
	ColoPrint (Agentia)	18	Microarray	Prognostic of recurrence in stage I through III colon cancer	Not recommended National Comprehensive Cancer Network (2021b)
	ColDx (Almac Diagnostic Services)	634	Microarray	Prognostic of recurrence in stage II colon cancer	Not recommended National Comprehensive Cancer Network (2021b)
Solid tumours	Caris Molecular Intelligence (Caris Life Sciences) CARIS, (2021)	HLA genotyping (55 fusions and 3 variant transcripts mostly associated with cancer and response to certain drugs)	RNA-seq	Treatment recommendations based on a multi-level molecular (DNA, RNA and protein) profiling of locally advanced or metastatic cancer	NICE advice MIB120^d^ NICE (2017)
Uveal melanoma	Decision DX-UM (Castle Biosciences) Aaberg et al. (2020)	15	RT-PCR	Predictive of 5-year metastatic risk guiding surveillance	NCCN National Comprehensive Cancer Network (2022)

^a^ The searches were conducted on databases (e.g., PubMed) and websites of guideline producers (e.g., NICE), leading authorities (e.g., The Centers for Medicare and Medicaid Services) and health technology assessment agencies and the lists are non-exhaustive. Additional commercially available gene expression signatures for early breast cancer: Rotterdam signature (Veridex, Johnson & Johnson), OncoMasTR, BluePrint (Agentia), Breast Cancer Index (Biotheranostics; complements histologic grading), Mapquant DX (also known as Genomic Grade Index; Ipsogen; complements histologic grading), MammaTyper (Biontech; RT-PCR, as an alternative to immunohistochemistry for quantification of HER2, ER, PR, and marker of proliferation Ki-67 used in molecular subtyping), Curbest 95GC, Breast Ca Gene Expression Ratio (Theros H/I), BreastNext, BreastOncPX, BreastPRS, combimatrix breast cancer profile, eXagen, Invasiveness Signature, Insight DX, breast cancer profile, MammoStrat, NexCourse Breast IHC4, NuvoSelect eRx 200-Gene Assay, Randox Assay, SYMPHONY, genomic breast cancer profile, TargetPrint, TheraPrint, The 41-gene signature assay, THEROS, Breast Cancer Index. Commercially available assays for other cancers: Lung RS, Oncomine Dx Target Test (lung), ExoDx Prostate EPI-CE, Afirma (thyroid), ThyroSeq v3 Genomic Classifier, DecisionDx-Melanoma (Castle Biosciences), MYPATH, Melanoma assay (Myriad Genetics), Pigmented Lesion Assay (DermTech), MyPRS, Plus GEP70 (multiple myeloma), MMprofiler (multiple myeloma), ResponseDX (cancer of unknown origin), Pathwork Test Kit (cancer of unknown origin), Oncofocus (cancer of unknown origin), CancerTypeID (cancer of unknown origin), miRview (cancer of unknown origin), RosettaCX, cancer origin test, OneRNA (RNA-seq, based test assisting in cancer treatment selection regardless of disease site). Other commercially available assays: AlloMap (heart transplant), TruGraf (kidney transplant), Corus CAD (obstructive coronary artery disease), SGES/CardioDX (coronary artery disease), PredictSure-IBD.

^b^ ESMO (level of evidence, grade of recommendation).

^c^ Direct mRNA, labelling with fluorescent probes and measuring with nCounter Digital Analyser.

^d^ A Medtech Innovation Briefing (MIB) is not NICE, guidance but an objective description of the technology to aid clinical decision-making.

RT-PCR, Reverse transcriptase polymerase chain reaction; HR+, Hormone Receptor-positive; HER2-, Human Epidermal growth factor Receptor 2-negative; LN ≤ 3, Lymph Node-negative or up to three-positive; T ≤ 5, Tumour size up to 5 cm; ASCO, american society of clinical oncology, ESMO, European society for medical oncology; NCCN, national comprehensive cancer network; NICE, the national institute for health and care excellence; RNA-seq, RNA, sequencing.

### 3.2 Development of Oncotype DX

The development of Oncotype DX was a gradual process involving the use of data from large clinical studies and diligent address of issues (Supplementary Box S2). Due to the remarkable molecular diversity of breast tumours (Perou et al., 2000), numerous clinical and immunohistochemical biomarkers and their combinations had failed to guide treatment decisions (Hayes, 2000). Moreover, previous attempts to identify predictive and prognostic gene expression signatures were based on single studies which were neither standardised nor reproducible. The 21-gene signature in Oncotype DX was derived from a set of 250 genes which was selected from well-designed studies and public databases utilising microarrays (Paik et al., 2004). The 250 candidate genes were narrowed down to 21 through three independent clinical studies including almost 500 patients who received adjuvant hormonal treatment plus chemotherapy or hormonal treatment alone (Paik et al., 2003; Cobleigh et al., 2005). An algorithm was developed to produce a continuous variable, the Recurrence Score (RS) based on the expression of these genes, which is comprehensible by clinicians and stratifies patients into high and low risk groups for distant recurrence within 10 years of surgery (Paik et al., 2004). RS showed remarkable statistically significant prognostic ability and predictive ability and has been extensively validated in large prospective randomised clinical trials and real-world data from population-based registries (Paik et al., 2004; Nitz et al., 2017; Sparano et al., 2018; Syed, 2020). Further analyses of these studies have identified that pre-menopausal women would benefit from the addition of clinical factors (age, tumour size, and histologic grade) along with RS for shaping management strategies (Hunter and Longo, 2019; Sparano et al., 2019).

Oncotype DX and the Decipher Genomic Classifier (21 and 22 expressed genes, respectively) have been shown to be cost-effective approaches for guidance of treatment decisions (Lobo et al., 2017; Berdunov et al., 2022). This results from a combination of test accuracy in reducing unnecessary toxic treatments such as chemotherapy and radiation while not excluding patients from beneficial treatments (Lux et al., 2022).

Following on from the success of the oncotype Dx for early breast cancer, the Oncotype DX Genomic Prostate Score has been developed on the same principles and similar processes (Supplementary Box S3). It aims to prevent unnecessary surgery and radiation by stratifying patients into low-risk and aggressive disease. However, there are no large prospective studies to validate the prognostic performance of the assay for clinical outcomes (Eggener et al., 2019; Brooks et al., 2021). Attempts to identify a gene expression signature prognostic of prostate cancer are based on tissue samples derived from needle-core biopsies and the limited amount of tissue may be a constrain to characterise heterogeneity (Supplementary Box S3).

Disease heterogeneity is a major caveat in the design of diagnostic biomarkers. Inter-assay comparisons revealed discordance in prognostic performance of gene expression-based tests for stratification of patients with early breast cancer (Varga et al., 2019; Abdelhakam et al., 2021; Buus et al., 2021). These discrepancies may derive from the diversity in gene sets, methodology and algorithms and design of studies. Of note, there is only minor overlap of genes among predictive tests (Supplementary Table S1). Heterogeneity in gene composition reflects the variety of molecular mechanisms involved in disease progression and it may not necessarily influence prognostic ability. Currently, a prospective study is investigating the clinical validity of Curebest 95GC, a microarray-based measurement of the whole genome in tumour tissues (Naoi et al., 2021). The results are anticipated to shed light on the number of transcripts required for stratification of patients with early breast cancer. However, an increased number of genes may be a major obstacle in the development of a cost-effective marker and mechanistic studies could assist with reducing the number (Gliddon et al., 2018).

### 3.3 Multi-layered heterogeneity at the tissue level

Heterogeneity at the tissue level is multi-layered and not confined to the oncogenic cells. Neoplastic cancer cells are nurtured by neighbouring stromal cells comprising the tumour microenvironment (TME). A diverse community of tumour infiltrating immune cells is a major component of the stromal microenvironment exerting both beneficial and detrimental effects (Hanahan and Coussens, 2012). Growing evidence shows that quantification of the proportion of leucocyte subsets can assist in prognosis and therapy choice (Gentles et al., 2015). Traditional methods such as immunohistochemistry and flow cytometry can identify a limited number of pre-defined cell populations but fail to discriminate unknown or closely related phenotypes.

By contrast, gene expression profiling coupled with computational algorithms can characterise cell composition of complex tissues (Finotello and Trajanoski, 2018; Xu et al., 2021). Many tools have been developed based on two main methods:

• *in silico* deconvolution (CYBERSORT, TIMER, EPIC, quanTIseq, DeconRNAseq, PERT, DSA, MMAD, ssKL); and
• gene set enrichment analysis (xCell, TIminer, MCP-counter) (Finotello and Trajanoski, 2018).

Deconvolution is based on a linear model of the expression of a gene in the different cell types. Digital dissection of the tumour into the relative fractions of cell types is estimated against a library of cell-specific expression signatures (reference signature or matrix). The matrix appears rigid considering the diversity of infiltrating immune cells (extend, type, activation status, interactions, and closely related cells) among tissues and cancer stages (Hanahan and Coussens, 2012; Newman et al., 2015). Refinement of the gene set enrichment method attempts to circumvent the issue by producing enrichment scores based on the expression levels of a set of cell-type-specific marker genes by analysing various data sources (Aran et al., 2017). However, performance is poorer in real mixtures compared to simulated mixtures and statistical significance is not reported for prediction of cell abundance (Newman et al., 2015; Aran et al., 2017). Deconvolution algorithms which simultaneously estimate relative cell fractions and produce a matrix of expression profiles have been developed (MMAD, DSA, ssKL, ssFrobenius, and deconf), but they are flawed by mathematical complexity and a limited ability to quantify a higher number of immune cells (Finotello and Trajanoski, 2018). Although several studies have tested these computational approaches in simulated samples and publicly available datasets showing good performance, evidence about their clinical validity is scarce (Desmedt et al., 2018; Newman et al., 2019; Waks et al., 2019; Craven et al., 2021).

It is unclear if gene signature enrichment and deconvolution approaches accurately portray the complexity of cellular heterogeneity in cancer samples and more work is warranted before testing in clinical settings. Definition of reference expression profiles is a fundamental caveat allowing for the identification of only a few dozens of cell types which may not reflect all heterogenic subsets in tumours. The effort should probably be on revealing hallmark phenotypes with prognostic and predictive capability in clinical settings to populate the reference matrix or marker gene-sets. For instance, the role of exhausted (increased PD-1 expression) CD8^+^ tumour infiltrating lymphocytes is well established in melanoma, renal and non-small cell lung cancer and it has guided the use of immune check point inhibitors (ICIs) (Sade-Feldman et al., 2018; Thommen et al., 2018; Young et al., 2018; McLane et al., 2019). Tumour-associated macrophages are another interesting group of cells because of their abundance in the tumour microenvironment. Unravelling of the complex subpopulations has shown that the classical categorisation to M1 and M2 polarised macrophages is an oversimplification of their crucial role in cancer regulation (Mantovani and Longo, 2018; Duan and Luo, 2021; Xiang et al., 2021).

## 4 Sepsis

### 4.1 Dynamic heterogeneity: The sepsis paradigm

Immune response to infection is initiated by a “genomic storm” of both pro-inflammatory and anti-inflammatory cytokines expressed concomitantly (Nakamori et al., 2020). In sepsis there is acute cellular reprogramming and failure to restore balance between immune activation and suppression can present with life-threatening organ dysfunction (Singer et al., 2016; van der Poll et al., 2017). Gene expression studies have revealed remarkable heterogeneity in sepsis due to host parameters (e.g., genomic variation and co-morbidities), source of infection and stage of illness. This may explain, at least partly, the reason for the failure of numerous promising therapeutic agents in clinical trials (Marshall, 2014; Davenport et al., 2016; Peters-Sengers et al., 2022). The definition of sepsis has also been revised several times, and each definition can dramatically alter the composition of cohorts that are included in studies (Johnson et al., 2018). This can also negatively impact model development, particularly where retrospective data collection is required or data are pooled across studies (Sauer et al., 2022).

To address individual variation in the response to sepsis, a multi-layered approach, including at the molecular level, for stratification in treatment subgroups is required. As a proof-of-concept, machine learning algorithms which classify patients based on routine clinical data have been shown to accurately predict clinical outcomes and sepsis onset (Komorowski et al., 2018; Seymour et al., 2019; Fleuren et al., 2020). Machine learning is a very powerful tool for harnessing large-scale data with the aim of identifying predictive biomarkers (Zhang et al., 2021). The use of diverse methods analysing transcriptomic data in various conditions has been previously reviewed (Vadapalli et al., 2022). Appreciation of common pitfalls and focus on interpretable findings has transformed these complex computational approaches into comprehensive tools (Sidak et al., 2022; Whalen et al., 2022). However, despite our increased understanding of sepsis pathogenesis with new technologies, translation of research knowledge to improvements in clinical practice has been exceedingly difficult.

### 4.2 Stratification of patients with sepsis

Transcriptomic-based real-time subclassification of patients has been developed and validated in individual studies (Table 2). The Knight group investigated gene expression profiles in peripheral blood leukocytes of patients on Intensive Care Units (ICU) with faecal peritonitis and community acquired pneumonia (Davenport et al., 2016). They proposed two sepsis phenotypes associated with prognosis. Genes comprising the sepsis response signature (SRS) demonstrated significant overlap between the two sources of infection and with trauma patients, while gene expression and SRS membership changed temporally (Burnham et al., 2017). Single-cell multi-omics evaluation showed that an immature immunosuppressive population of neutrophils together with enrichment in the IL-1 pathway are the biological underpinnings of the SRS1 group who experienced increased early mortality (Kwok et al., 2022). In contrast, the immuno-competency of the SRS2 endotype was compromised by corticosteroids in a randomised clinical trial which showed an association between hydrocortisone use and higher mortality in the SRS2 group but not in the SRS1 group (Antcliffe et al., 2019). The SRS investigators upgraded their classifier to the SepstratifieR framework which can be applied to multiple infecting pathogens and data accruing from different platforms (e.g., RNA-seq and RT-qPCR). SepstratifieR utilises expression levels of signature genes, including an extended 19-gene set expected to be robust to technological variation, to align samples to a corresponding reference map and returns the SRS endotype and a severity score (SRSq). SRSq reflects immune deregulation and has the advantage of modelling patients as a continuum which is a better descriptor of molecular profiles compared to classes (Cano-Gamez et al., 2022).

**TABLE 2:** A summary of studies identifying gene expression signatures to classify patients with critical illness due to infection.

First author, year of publication	Study design	Condition/Infection	Sample type	Sample size	Platform	Classifier training approach	No of DEG	Biological functions/pathways	Patient stratification	Gene signature/classifier
Davenport et al. (2016)	Prospective observational	CAP	Peripheral blood leukocytes	Discovery: 265	Illumina Human-HT-12 version 4 Expression BeadChips	Unsupervised hierarchical cluster analysis, sparse regression variable selection	3,080	T-cell activation, cell death, apoptosis, necrosis, cytotoxicity, phagocyte movement	SRS1: immuno-compromised and high mortality and SRS2: immuno-competency and low mortality	DYRK2, CCNB1IP1, TDRD9, ZAP70, ARL14EP, MDC1, ADGRE3 (Davenport signature)
				Validation: 106						
Burnham et al. (2017)	Prospective observational	Faecal peritonitis (FP)	Peripheral blood leukocytes	Discovery: 67	Illumina Human-HT-12 version 4 Expression BeadChips	Unsupervised hierarchical cluster analysis, sparse regression variable selection	1,075	Cell death, apoptosis, necrosis, T-cell activation, endotoxin tolerance	SRS1, SRS2, SRS1_FP and SRS2_FP	Membership assignment based on expression of the Davenport signature, plus a new six-gene signature for FP
				Validation: 53						
Cano-Gamez et al. (2022)	Prospective observational	CAP, FP and health	Peripheral blood leukocytes and whole blood	Training: 909	Microarray	Diffusion maps and random forest	7,171	innate immune pathways, glycolysis, T-cell activation	SRSq: 0–1 with lower values indicating a patient is transcriptionally closer to health and higher values indicating similarity to SRS1	Davenport genes and FBXO31, BMS1, SH3GLB1, TTC3, USP5, UBAP1, PGS1, MRPS9, THOC1, NAT10, DNAJA3, SLC25A38
				Test: 2,355	RNA-seq					
					RT-PCR					
Scicluna et al. (2017)	Prospective observational	Probable or definite infection	Whole blood	Discovery: 306	Affymetrix Human Genome U219 96-array plates	Hierarchical consensus clustering and random forest	9,699	PRR and cytokine signalling, adaptive immune functions, heme biosynthesis, lymphocyte signalling, antigen presentation	Mars1-4 with Mars1 having highest mortality and immunosuppression, Mars3 being low risk and Mars4 having variable mortality among the cohorts	140-gene set -> BPGM:TAP2 (Mars1) GADD45A:PCGF5 (Mars2) AHNAK:PDCD10 (Mars3) IFIT5:GLTSCR2 (Mars4)
				Validation1: 216						
				Validation2: 265						
Scicluna et al. (2015)	Prospective observational	CAP	Whole blood	Discovery: 101	Affymetrix Human Genome U219 96-array plates	Differential gene expression analysis of CAP vs. no-CAP, followed by nearest shrunken centroid classification	2,459	eIF2 signalling, T-cell receptor signalling and mTOR signalling	N/A	78-gene set -> FAIM3:PLAC8
				Validation: 70						
Wong et al. (2009) and Wong et al. (2011)	Prospective observational	Septic shock	Whole blood	Discovery: 98	Affymetrix Human Genome U133 Plus 2.0 GeneChip	Differential gene expression analysis, unsupervised hierarchical clustering, analysis functional enrichment and K-means clustering.	6,934	Adaptive immunity and glucocorticoid receptor signalling	Subclass A, B and C with A having higher illness severity and mortality and repressed gene expression patterns	100-gene set
				Validation: 82						
Wong et al. (2015)	Retrospective and prospective observational	Septic shock	Whole blood	Discovery: 168	NanoString nCouter	100-gene set reformulated as gene expression mosaics (GEDI) and composite variability scores	n/a	Adaptive immunity and glucocorticoid receptor signalling	Subclass A and B with A having worse outcomes and lower Gene Expression Score (GES)	100-gene set summarised as an expression mosaic, GEDI
				Validation (inter-assay): 132						
Sweeney et al. (2015)	Meta-analysis of publicly available datasets	SIRS/trauma vs. sepsis/infection	Whole blood and buffy coat	Discovery: 9 cohorts (n = 663)	Microarrays^a^	Gene filtering by effect-size and Fisher’s method using leave-one-data set-out multi-cohort analysis, followed by greedy forward search modelling	82	Downstream of IL-6 and JUN	Infection z-score derived from the geometric mean of the 11-gene set with higher scores for infected patients which peaked within 1 day of diagnosis and declined over time similarly in infected and non-infected patients	Sepsis MetaScore (SMS): CEACAM1, ZDHHC19, C9orf95, GNA15, BATF, C3AR1, KIAA1370, TGFBI, MTCH1, RPGRIP1, HLA-DPB1
				Validation: 15 independent cohorts^b^						
Sweeney et al. (2018a)	Retrospective analysis	Bacterial sepsis	Whole blood	Discovery: 14 datasets (*n* = 700)	Microarrays^c^	Iterative clustering algorithm (COMMUNAL) combining K-means and consensus PAM clustering, significance analysis for microarrays (SAM), greedy forward search then multinomial logistic regression on the separation scores.	n/a	IL-1 receptor, PRR activity, complement activation, adaptive immunity and interferon signalling, platelet degranulation, glycosaminoglycan binding, coagulation cascade	Inflammopathic cluster (high mortality), Adaptive (lower mortality) and Coagulopathic (high mortality and older patients)	33-gene set
				Validation: 9 datasets (*n* = 600)						
McHugh et al. (2015)		Sepsis vs. non-infective systemic inflammation	Whole blood	Discovery: 74 cases vs. 31 controls (*n* = 105)	Affymetrix Human Exon 1.0 ST arrays (modified) and RT-PCR for the validation cohorts	Recursive feature elimination support vector machines and backwards elimination random forests, followed by greedy search of log gene-pair ratios	n/a	Innate immunity	SeptiScore: low values correlated with low sepsis probability (cut-off of 4)	SeptiCyte Lab: PLA2G7/PLAC8 and CEACAM4/LAMP1 ratios
				Validation: 5 cohorts (*n* = 345) from MARS						

^a^ Affymetrix Human Genome U133 Plus 2.0 Array (GPL570), Illumina Human-HT-12, version 4 Expression BeadChips (GPL10558) and Illumina HumanHT-12, V3.0 expression beadchip (GPL6947).

^b^ *n* = 218 from the Glue Grant sorted-cells cohort, *n* = 215 from three longitudinally sampled cohorts, *n* = 446 from eight cohorts comparing infection vs. health, *n* = 274 of a cohort comparing bacterial infection vs. autoimmune inflammation or health.

^c^ GPL96, GPL570, GPL571, GPL6106, GPL6244, GPL6947, GPL10332, GPL10558, and GPL13667.

DEG, Differentially expressed genes; CAP, Community acquired pneumonia; SRS, Sepsis response signature; FP, Faecal peritonitis; RNA-seq, RNA sequencing; RT-PCR, Reverse transcriptase polymerase chain reaction; PRR, Pattern recognition receptor; Mars, Molecular diagnosis and risk stratification of sepsis; eIF2, eukaryotic initiation factor 2; mTOR, mechanistic target of rapamycin; GEDI, Gene expression dynamics inspector; SIRS, Systemic inflammatory response syndrome; IL, interleukin; COMMUNAL, Combined mapping of multiple clUsteriNg algorithms.

The Molecular Diagnosis and Risk Stratification of Sepsis (MARS) project identified four endotypes (Mars1–4) in patients with sepsis admitted to ICU in the Netherlands (Scicluna et al., 2017). Biomarkers for each endotype were derived from a 140-gene expression signature (Table 2). The authors proposed that patients classified as Mars1 were the most clinically relevant group with consistently increased mortality. Comparisons with the SRS revealed an overlap between the low-risk groups SRS2 and Mars3, but not the expected enrichment of Mars1 patients within SRS1 (Scicluna et al., 2017; Cano-Gamez et al., 2022). One explanation could be the primarily leukocyte-based training data for SepstratifieR differing from the whole blood-derived RNA used for MARS signatures. The differences could also be attributed to variation in populations utilised for classifier development, technical procedures, bioinformatics analysis and study design (Table 3). At the gene level though, similarities in differential expression and active pathways were observed. Moreover, classification of MARS patients into SRS endotypes showed that SRS1 had a higher proportion of septic shock and elevated Sequential Organ Failure Assessment (SOFA) scores, but not increased mortality, reflecting the presence of unobserved variables preventing the severe sequelae of sepsis (Cano-Gamez et al., 2022).

**TABLE 3:** Differences between MARS and SRS discovery cohorts.

Parameter	MARS discovery cohort (*n* = 306)	SRS discovery cohort (*n* = 265)
Demographics	Netherlands	United Kingdom
Top comorbidities	None (41%)	Respiratory insufficiency (48%) and cardiovascular compromise (45%)
Source of infection	Multiple with 42% lung and 26% abdominal	Lung
SOFA score - Shock, %	6%–35%	6%–30%
AKI	43%	20%
Length of ICU stay, days	4	7
28-day mortality	28%	21%
Sample collection	PAXgene blood RNA tubes	Leukocyte separation at bedside (LeukoLOCK)
Microarray platform	Affymetrix (49,386 probes)	Illumina (47,231 probes)

Mars, Molecular diagnosis and risk stratification of sepsis; SRS, Sepsis response signature; SOFA: Sequential organ failure assessment; AKI, Acute kidney injury; ICU, Intensive care unit.

Paediatric patients with septic shock were categorised into three groups based on a 100-gene set microarray-derived signature (Wong et al., 2009; Wong et al., 2011). Two (A and B) of the three subclasses were identified with the use of a different platform for mRNA quantification (NanoString nCounter) which has the potential for clinical application due to its decreased turnaround time and cost (Wong et al., 2015). The authors noted that subjects in subclass B and C demonstrated similar clinical phenotypes, whereas subclass A patients had poorer outcomes. The previously reported association between mortality and corticosteroid use among patients of a specific endotype was also observed, but this time within the subclass with increased mortality (subclass A, Wong et al., 2015). Interestingly, when the Mars signature was applied to the original paediatric population, only three of the four endotypes were stably recognised and there was no association between endotype categorisation and mortality (Scicluna et al., 2017). The search for prognostic biomarkers in paediatric septic shock has led to the development of the paediatric Sepsis Biomarker Risk Model (PERSEVERE), which is a predictive tool of mortality and disease severity (Jacobs et al., 2019; Wong et al., 2019). A panel of 117 gene probes possibly associated with outcome in children with septic shock was used to select 12 genes with a protein product which had a mechanistic role in immune responses to infection and was readily measured in serum. Classification and regression tree analysis reduced the number of proteins to five and selected age among various clinical parameters as the best combination of factors to predict 28-day mortality (Wong et al., 2012). PERSEVERE incorporates the protein products of those five genes and has been tested as a predictor of sepsis-related organ dysfunction in various cohorts (Wong et al., 2016; Yehya and Wong, 2018; Stanski et al., 2020; Al Gharaibeh et al., 2022; Atreya et al., 2022). Clinical utility is yet to be decided through large prospective validation studies. Utilization of proteins identified through gene expression exploratory studies may achieve better reproducibility among cohorts, but correlation of mRNA and protein product is affected by various biological and technical parameters and clinical translation is yet to be decided.

### 4.3 Use of publicly available datasets and validation

The aforementioned unsupervised clustering studies (Table 2) defined novel molecular subgroups in sepsis and produced data-driven classifiers with potential for clinical implementation. Although such approaches are the foundation of precision medicine, results are often non-reproducible because they accrue in a method-specific computational manner and/or from underpowered sample sizes. The availability of high-dimensional data from various studies in public databases and meta-clustering techniques have allowed the development of transcription-based models with improved representation of disease and population heterogeneity. A large pool of bacterial sepsis transcriptomic datasets (23 datasets; *n* = 1,300) identified three clusters which were descriptive of underlying molecular pathways, the Inflammopathic, the Adaptive and the Coagulopathic (Table 2). Comparisons with previously published signatures showed that the inflammopathic cluster tended to overlap with the paediatric septic shock subclass B and SRS1 and the Adaptive cluster was associated with SRS2 (Sweeney et al., 2018a). Identification of the same group of sepsis patients in independent studies with separate techniques supports the existence of molecular subtypes. The addition of a third cluster in a bigger study underscores the importance of utilising large public datasets. In a community-based approach, three independent teams built four separate models to predict mortality in sepsis using all available gene expression datasets (Sweeney et al., 2018b). Despite common data inputs, there was little overlap in predictive genes between groups due to differences in analytical approaches. Still, the model performances were broadly similar. Moreover, the combination of gene expression-based predictors with routine clinical parameters was shown to improve prognostic accuracy (Wong et al., 2014; Scicluna et al., 2017; Sweeney et al., 2018b).

The predictive performance of candidate biomarkers attempting to distinguish between the presence and absence of infection in critically ill patients has historically been suboptimal (Pierrakos and Vincent, 2010; Wacker et al., 2013). An informative biomarker consisting of a gene expression ratio has been proposed to assist in discriminating between community acquired pneumonia (CAP) and non-CAP patients, but its relatively low negative predictive value precludes it from being a stand-alone diagnostic test (Scicluna et al., 2015). Similarly, the FDA approved SeptiCyte LAB (Immunexpress, Seattle, WA), which provides a score based on the expression of four genes, is intended to be used in conjunction with clinical factors and clinical judgement to distinguish patients with sepsis from non-infective systemic inflammation within 24 h of ICU admission (McHugh et al., 2015). Different studies evaluating the discriminative power of this novel biomarker have produced conflicting results (McHugh et al., 2015; Zimmerman et al., 2017; Koster-Brouwer et al., 2018). Comparison of three scores aiming to distinguish between the presence or absence of infection in critically ill patients (FAIM3:PLAC8, SeptiCyte LAB and MetaScore or SMS) demonstrated similar performance with some superiority of the SMS (Table 2) when applied to a different cohort of patients (Sweeney and Khatri, 2017; Maslove et al., 2019). The absence of gold standard reference test dictated the use of strict criteria to define cases and controls for a supervised analytical approach for classifier development (Table 2). As a result, the discovery cohort cannot mirror the wide spectrum of heterogeneity which is inherent in sepsis patients. It is likely that leveraging of clinical and technical heterogeneity seen in larger publicly available datasets and extensive validation may help in ameliorating limitations regarding generalisability.

Transcriptomic and genomic samples are collected during most clinical trials in cancer and other diseases (NIH, 2022). Their aim is to increase our understanding of molecular mechanisms. Investigators are not obliged to submit transcriptional data deriving from interventional clinical trials to public databases unless they are presented in a publication. Hence, a plethora of interesting data may become available later or never. Clearly, it is important for investigators to deposit data from their studies in a standardised format into publicly available databases as such democratisation of data undoubtedly accelerates the pace of progress. We think that adequate progress from the translational to the clinical stage can be achieved with combination of data from different populations and to this purpose investigators should be assisted in processing their raw data early and prompted to deposit them in public databases.

### 4.4 Timing of sampling

**FIGURE 1:**
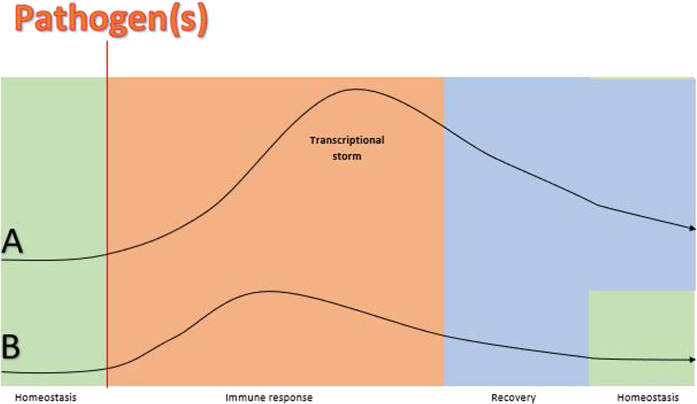
A theoretical schematic comparison of the size of gene expression trajectory before, during and after sepsis vs. gene expression response before, during and after the same infection but without sepsis. Lines **(A)** and **(B)** represent gene expression responses to a pathogen(s) in a patient with sepsis and without sepsis, respectively. The homeostasis balance (horizontal part of the lines) is disturbed in both cases by pathogen(s) but gene expression changes during the immune response phase are larger and more delayed (transcriptional storm curb) in the patient with sepsis **(A)** compared to the patient without sepsis **(B)**. The transcriptional storm represents hyper-inflammation and immunosuppression pathways which reflect the immune dysregulation in sepsis and result in organ damage (Nakamori et al., 2020). The onset of symptoms is not pointed in the diagram because the transcriptional response precedes symptomatology and this interim probably varies among individuals (Lukaszewski et al., 2022). Also, recovery is more prolonged in sepsis ans return to homeostasis may not achieved in some patients (Prescott and Angus, 2018). Findings of ongoing studies will shed light on the validity of the proposed model (Fish et al., 2022).

Although 80% of the blood transcriptome shows differential expression in critical illness, immune responses demonstrate significant commonality leading to a remarkable overlap in expressed genes in all-cause inflammation, regardless of the presence of an infection or not (van der Poll et al., 2017). A multicohort analysis of publicly available datasets showed that there is a small proportion of distinct genes in patients with sepsis compared to patients with a non-infective critical condition in samples obtained within 48 h of admission (Sweeney et al., 2015). These findings highlight the common trajectory of the transcriptional storm that settles down during recovery underscoring the importance of time-course-based approaches (Sweeney and Wong, 2016). Gene expression signatures which predict infection have been identified in the blood of hospitalised patients up to 5 days prior to onset of symptoms and/or diagnosis (Johnson et al., 2007; Cobb et al., 2009; Sweeney et al., 2015; Yan et al., 2015; Lukaszewski et al., 2022). These findings highlight the molecular events which occur before disease symptomatology. If the immune response is not successful in clearing the pathogen(s) during this period, more robust measures are deployed leading to a transcriptional storm (Figure 1).

Tests based on gene expression thus describe “the moment in time” which has the potential for guiding targeted therapies and personalised management (van der Poll et al., 2017). However, there is no way to match the expressed molecular moment to the exact point of the disease (Figure 1) because the duration of each stage varies significantly. As an example, many groups put their efforts into identifying a classifier within 24 h of ICU admission. We may assume that this is located within the transcriptional storm space, but we cannot say whether it is in the beginning, middle, end of the curve or even within the pre-disease space. The point of symptom onset relative to the infection point potentially varies among individuals and so does presentation and admission time. Hence, despite the efforts of time-based approaches, sampling time can be defined only clinically and not objectively across the gene expression course, i.e. “one fits all” is unlikely to succeed. Challenge studies with controlled infection and longitudinal designs could shed more light on the importance of defining timing of sampling, but are complex to perform and expensive, and need to have a careful ethical framework.

### 4.5 Biomarkers for sepsis in the pipeline

There are few promising biomarkers currently in the pipeline. A combination of three non-overlapping signatures identified from a multi-cohort analysis (Sweeney et al., 2015; Sweeney et al., 2016; Sweeney et al., 2018b) has led to TriVerity (formerly known as InSepTM HostDxTMSepsis and Inflammatix) (Mayhew et al., 2020). This 29-gene expression-based test with a turnaround time less than 30 min is expected to identify the presence, type (bacterial or viral) and risk of mortality of infection (Mayhew et al., 2020; Bauer et al., 2021; Safarika et al., 2021; Brakenridge et al., 2022; Galtung et al., 2022). A Point-of-Care Test claiming to distinguish bacterial from viral infections in children is in its infancy (Pennisi et al., 2021). It is based on the expression of two genes (IFI44L and FAM89A) which emerged from a microarray-based study in almost 500 febrile children (Herberg et al., 2016; Kaforou et al., 2017). There is a repertoire of promising findings in children with infections such as *tuberculosis*, bacterial pneumonia, rhinovirus and respiratory syncytial virus (RSV) and the transfer of transcriptomics knowledge to routine clinical care may be seen in the near future (Mejias et al., 2021). Investigators have also adapted a mechanistic-orientated approach to select a set of genes with known correlation with sepsis outcome instead of a crude exploration of bulk RNA (Chen et al., 2022; Kreitmann et al., 2022), but further consideration of this is beyond the scope of this review.

## 5 Considerations for transcriptome biomarker data analysis

The promise offered by the transcriptome in diagnosing and predicting disease status and progression is exemplified by the cancer and sepsis studies discussed. Yet relatively few RNA-based genetic tests have regulatory approval for clinical use (Table 1). This illustrates the challenges when gaining robust insights from such complex data—not least of which includes the analytical approaches that might be taken. Though the costs of sequencing continue to decrease, the number of samples in individual transcriptomic studies tend to measure in the hundreds at most, in comparison to thousands of measured RNA molecules (Levy and Myers, 2016). The chosen statistical and machine learning methods employed to produce predictive models vary greatly across studies. Table 2 provides an indication of the variety of techniques employed to classify patients, in just one clinical context. Classification approaches used include unsupervised clustering, iterative or otherwise, regression analyses, tree-based classification methods, functional enrichment and variable selection, among others. Often a combination of these methods are employed. Sweeney et al. (2018b) demonstrate this problem of choice acutely with their community-based modelling of the same data sets. Four attempts were made across three institutions to predict sepsis prognosis, yielding different models that performed similarly but had few overlapping genes. Correlations of ranked sample scores across research groups were also moderate at best. Interestingly, on average the ensemble model did not substantially differ from the individual models—suggesting some form of plateau on classification accuracy had been reached.

The choice of analysis may also be guided by the final format that the test will take in the clinic. Here the medical need, timing of the test and costs should be considered. The proposed tests listed in Tables 1, 2 include RT-PCR assays, microarrays, Nanostring and RNA-seq methods. For sepsis where classification tests might favour rapid turnaround time, assays such as RT-PCR and Nanostring might be favourable as they yield results in a matter of hours (Wong et al., 2015). Other tests might be preferred where longer timeframes are acceptable. These might prove more cost-effective at measuring many genes, or provide robust results with convenient clinical samples such as FFPE tissue. The studies described all present a refined panel of genes or proteins as input for their classifiers, but the extent of refinement should be determined by the final assay choice for use in the clinic.

Notably, some of the attempts to apply tests to new populations find further model training is required, including the addition of more genes (Burnham et al., 2017; Cano-Gamez et al., 2022). This is perhaps to be expected given the heterogeneity of human samples and the complexity of the clinical problems. The model for Oncotype DX, approved for clinical use, was ultimately derived from pooling three clinical trials’ results (Paik et al., 2014). In the case of sepsis, attempts to use publicly available data to improve robustness may similarly prove fruitful (Sweeney et al., 2018a; Sweeney et al., 2018b; Cano-Gamez et al., 2022). Likewise, more groups taking steps to ensure their analyses can be reproduced and applied to new populations, by sharing code and data, should also hasten this process (Heil et al., 2021).

Another common feature of the discussed models is their propensity for improvement by the addition or stratification of clinical variables (Sweeney et al., 2018b; Sparano et al., 2019). Where possible, routinely collected clinical variables should be incorporated early into analyses of transcriptomic data to improve the prospects of the classifier in validation studies.

Advanced machine learning methods offer the ability to flexibly model complex relationships in data. This property might be ideal when considering transcriptomics in complex clinical contexts. The flexibility may also come at a cost, in demanding greater numbers of samples than comparatively simpler methods (van der Ploeg et al., 2014). In a study comparing commonly used methods, neural network approaches failed to demonstrate superiority over regression-based analyses for classifying phenotypes from transcriptomic data (Smith et al., 2020). Another benefit of relatively parsimonious models lies in the abundance of established theory for calculating prospective study sample sizes (Riley et al., 2020). Prospective validation of a final model is essential for regulatory approval, and careful planning with realistic expectations of model performance is essential to improve the chance of success. Finally, many of the studies discussed focus on the discriminative ability of their classifiers, but lack any calibration measures for the predicted probabilities these models often estimate. These measures are vital if the models are to be used for clinical decision making (Van Calster et al., 2019). Aiming for good calibration as well as discrimination will also reduce the risk of model overfitting, thereby increasing the likelihood of prospective validation.

## 6 Discussion

**FIGURE 2:**
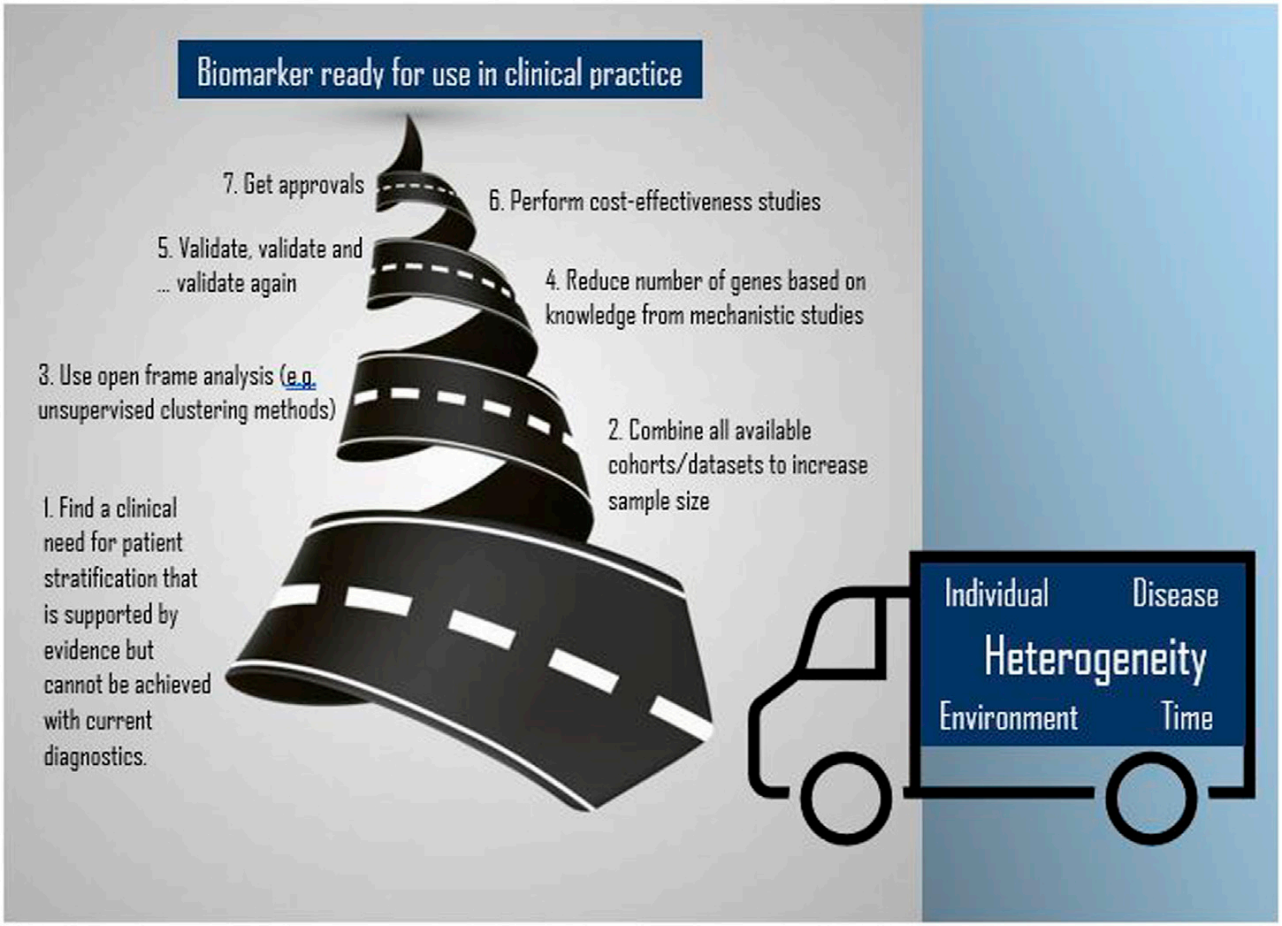
The road to implementing transcriptomics for biomarker development (spiral road image has been adapted from Vector: 13812147, standard licence reference No: 43565764).

The principles of traditional medicine should be upgraded to the tailored approaches of precision medicine. Gene expression-based tests are raw tools with a potential to be strategic for the diagnosis and management of patients. The transcriptome carries a massive amount of genetic and non-genetic information in time capturing cell, tissue, disease and host heterogeneity. The identification of transcriptional changes which initiate cell reprogramming carry fundamental prognostic and predictive value in cancer and sepsis diagnoses. The enormous pace of evolvement of technological and analytical methods precludes standardisation and increases variation which can be circumvented with the use of large amounts of data including those which are publicly available. The accruing plethora of data, not only from a single experiment, but also from the combination of multi-cohorts, instigates the use of open-frame approaches (e.g., unsupervised hierarchical clustering) and complex mathematical algorithms resulting in computational chaos. Hence, findings require vigorous confirmation with the use of conventional methods to monitor (e.g., reference genes) processes or validate results technically and clinically. To this point, study design is paramount. Discovery studies should aim to address specific and clinically relevant questions with patient stratification into prognostic and/or treatment groups through novel diagnostic tools which outperform standard practice. Validation should be driven by large prospective randomised clinical trials and population-based studies. Our increasing knowledge of the properties of the transcriptome and its regulators is our ally in all steps of the journey of developing improved diagnostic tools (Figure 2). Breast cancer and sepsis represent exemplars for the successful development of prognostic/predictive transcriptomics-based tests underscoring the optimisation of identified gene expression signatures into clinically relevant and feasible tests. Further development in both cancer and sepsis, and indeed in other disease areas, should herald a new era of clinical diagnostics and therapeutics.
