# Supplementary material for: Leveraging transcriptomics for precision diagnosis: Lessons learned from cancer and sepsis
Source: Front Genet. 2023 Mar 10;14:1100352. doi: 10.3389/fgene.2023.1100352 (PMC10036914; doi:10.3389/fgene.2023.1100352)
Supplement: Supplementary file 1 [file Table1.docx]

Supplementary Material

# Supplementary Table 1: Genes included in gene expression tests recommended by guideline producers

| Test | Genes |
| --- | --- |
| Oncotype Dx | MKI67^a^, STK15, BIRC5 ^a^, CCNB1 ^a^, MYBL2 ^a^, GRB7 ^a^, ERBB2 ^a^, ER, PGR ^a^, BCL2 ^a^, SCUBE2, MMP11 ^a^, CTSL2, GSTM1, CD68, BAG1 ^a^, ACTB^a,b^, GAPDH ^b^, RPLP0^a,b^, GUS^b^, TFRC^b^ |
| MammaPrint | AL080059, Contig 63649RC, LOC51203, Contig 46218RC, Contig 38288RC, AA555029RC, Contig 28552RC, FLT1, MMP9, DC13, EXT1, AL137718, PK428, HEC, ECT2, GMPS, Contig 32185RC, UCH37, Contig 35251RC, KIAA1067, GNAZ, SERF1A, OXCT, ORC6L, L2DTL, PRC1, AF052162, COL4A2, KIAA0175, RAB6B, Contig 55725RC, DCK, CENPA^a^, SM20, MCM6, AKAP2, Contig 56457RC, RFC4, DKFZP564D0462, SLC2A3, MP1, Contig 40831RC, Contig 24252RC, FLJ11190, Contig 51464RC, IGFBP5, IGFBP5, CCNE2, ESM1, Contig 20217RC, NMU, LOC57110, Contig 63102RC, PECI, AP2B1, CFFM4, PECI, TGFB3, Contig 46223RC, Contig 55377RC, HSA250839, GSTM3, BBC3, CEGP1, Contig 48328RC, WISP1, ALDH4, KIAA1442, Contig 32125RC, FGF18 |
| Prosigna PAM50 assay | FOXC1, MIA, KNTC2, CEP55, ANLN, MELK, GPR160, TIMEM45B, ESR1, FOXA1, ERBB2 ^a^, GRB7 ^a^, FGFR4, BLVRA, BAG1 ^a^, CDC20, CCNE1, ACTR3B, MYC, SFRP1, KRT14, KRT17, KRT5, MLPH, CCNB1 ^a^, CDC6, TYMS, UBE2T, RRM2 ^a^, MMP11 ^a^, CXXC5, ORCL6, MDM2, KIF2C, PGR ^a^, MKI67 ^a^, BCL2 ^a^, EGFR, PHGDH, CDH3, NAT1, SLC39A6, MAPT, UBE2C ^a^, PTTG1, EXO1, CENPF, CDCA1, MYBL2 ^a^, BIRC5, ACTB ^a,b^, RPLP0 ^a,b^, MRPL19^b^, SF3A1^b^, PSMC4^b^ |
| Endopredict | BIRC5 ^a^, UBE2C ^a^, DHCR7, RBBP8, IL6ST, AZGP1, MGP, STC2, CALM2^b^, OAZ1^b^, RPL37A^b^ |
| Breast Cancer Index | HOXB13:IL17BR and BUB1B, CENPA ^a^, NEK2, RACGAP1 and RRM2 ^a^ (Molecular Grade Index, MGI) |

*a: genes encountered in more than one signature*

*b: reference genes used for normalization*

# References

FILIPITS, M., RUDAS, M., JAKESZ, R., DUBSKY, P., FITZAL, F., SINGER, C. F., DIETZE, O., GREIL, R., JELEN, A., SEVELDA, P., FREIBAUER, C., MÜLLER, V., JÄNICKE, F., SCHMIDT, M., KÖLBL, H., RODY, A., KAUFMANN, M., SCHROTH, W., BRAUCH, H., SCHWAB, M., FRITZ, P., WEBER, K. E., FEDER, I. S., HENNIG, G., KRONENWETT, R., GEHRMANN, M. & GNANT, M. 2011. A new molecular predictor of distant recurrence in ER-positive, HER2-negative breast cancer adds independent information to conventional clinical risk factors. Clin Cancer Res, 17, 6012-20.

MA, X.-J., SALUNGA, R., DAHIYA, S., WANG, W., CARNEY, E., DURBECQ, V., HARRIS, A., GOSS, P., SOTIRIOU, C., ERLANDER, M. & SGROI, D. 2008. A Five-Gene Molecular Grade Index and <em>HOXB13:IL17BR</em> Are Complementary Prognostic Factors in Early Stage Breast Cancer. 14, 2601-2608.

PAIK, S., SHAK, S., TANG, G., KIM, C., BAKER, J., CRONIN, M., BAEHNER, F. L., WALKER, M. G., WATSON, D., PARK, T., HILLER, W., FISHER, E. R., WICKERHAM, D. L., BRYANT, J. & WOLMARK, N. 2004. A multigene assay to predict recurrence of tamoxifen-treated, node-negative breast cancer. N Engl J Med, 351, 2817-26.

PARKER, J. S., MULLINS, M., CHEANG, M. C. U., LEUNG, S., VODUC, D., VICKERY, T., DAVIES, S., FAURON, C., HE, X., HU, Z., QUACKENBUSH, J. F., STIJLEMAN, I. J., PALAZZO, J., MARRON, J. S., NOBEL, A. B., MARDIS, E., NIELSEN, T. O., ELLIS, M. J., PEROU, C. M. & BERNARD, P. S. 2009. Supervised risk predictor of breast cancer based on intrinsic subtypes. Journal of clinical oncology: official journal of the American Society of Clinical Oncology, 27, 1160-1167.

VAN 'T VEER, L. J., DAI, H., VAN DE VIJVER, M. J., HE, Y. D., HART, A. A., MAO, M., PETERSE, H. L., VAN DER KOOY, K., MARTON, M. J., WITTEVEEN, A. T., SCHREIBER, G. J., KERKHOVEN, R. M., ROBERTS, C., LINSLEY, P. S., BERNARDS, R. & FRIEND, S. H. 2002. Gene expression profiling predicts clinical outcome of breast cancer. Nature, 415, 530-6.
