# Supplementary material for: Leveraging transcriptomics for precision diagnosis: Lessons learned from cancer and sepsis
Source: Front Genet. 2023 Mar 10;14:1100352. doi: 10.3389/fgene.2023.1100352 (PMC10036914; doi:10.3389/fgene.2023.1100352)
Supplement: Supplementary file 2 [file DataSheet1.zip › SupplementaryMaterial_Box1.docx]

Supplementary Material

# Supplementary Box 1: Breast cancer in context

| - Early (or early stage invasive) breast cancer is localised in the breast with or without regional lymph node involvement and there is no distant metastatic disease (Harbeck et al., 2019). Around two thirds of breast cancer is ER+/PR+/HER2- (encoded by the ESR1, PGR and ERBB2 genes, respectively) (National Cancer Institute Surveillance Epidemiology and End Results Program (SEER), 2014-2018) and categorised as luminal A with good prognosis (Harbeck et al., 2019). Targeted therapies, such as endocrine blockers, have improved clinical outcomes and early breast cancer is curable in around 70% to 80% of patients. - Molecular subtyping of breast cancer is prognostic. It is mainly characterised by the expression of ER and HER2, but further molecularly distinct types have been characterised (Vieira and Schmitt, 2018). Perou and colleagues originally assumed that samples from the same tumour should have similar gene expression profiles which vary among different tumours. A 496-gene signature from 65 tissue samples including 22 paired samples (open surgery biopsy followed by treatment with doxorubicin and surgery after 16 weeks) was able to classify tumours which were clinically described as ER+ (luminal) and ER- with the latter being further clustered to HER2+, HER2- (basal) and normal tissue-like carcinomas (Perou et al., 2000). This gene subset was further optimised to the PAM50 signature distinguishing ER+ to luminal A (HER2-) and B (HER2+) and identifying the triple-negative/claudin low subtype (Cheang et al., 2015). In clinical settings, five surrogate subtypes are used which are defined through more traditional techniques such as histology and immunohistochemistry (Harbeck et al., 2019). - The molecular background of breast cancer has been extensively investigated with cutting-edge multi-omics technologies. As a result, an in-depth understanding of disease processes and methodological advancements have offered valuable insights which have been translated in clinical care. Utilisation of gene expression signatures as predictive biomarkers of adjuvant therapy benefit is exemplary. Chemotherapy reduces risk of recurrence by approximately one third with odds of severe toxicities (permanent disability, life-threatening or fatal side effects) being at least 2% to 3% in healthy women. Among patients with grade 1 (well-differentiated cells on histology) ER+/PR+/HER2-/LN- breast cancer treated with adjuvant endocrine therapy only 2% to 3% will benefit from the addition of chemotherapy balancing benefits with harms. By contrast, in a triple negative (ER/PgR/HER2) and LN+ scenario chemotherapy reduces recurrence risk by 15% to 20% (Harris et al., 2016). A clinical biomarker should be able to identify reliably this group of patients for whom the benefit of chemotherapy will not exceed 2% to 3% in order to recommend sparing of a potentially life-saving treatment. Alternatively, an ideal biomarker should also be able to predict the group of patients who will experience severe toxicities. |
| --- |

# References

CHEANG, M. C., MARTIN, M., NIELSEN, T. O., PRAT, A., VODUC, D., RODRIGUEZ-LESCURE, A., RUIZ, A., CHIA, S., SHEPHERD, L., RUIZ-BORREGO, M., CALVO, L., ALBA, E., CARRASCO, E., CABALLERO, R., TU, D., PRITCHARD, K. I., LEVINE, M. N., BRAMWELL, V. H., PARKER, J., BERNARD, P. S., ELLIS, M. J., PEROU, C. M., DI LEO, A. & CAREY, L. A. 2015. Defining breast cancer intrinsic subtypes by quantitative receptor expression. Oncologist, 20, 474-82.

HARBECK, N., PENAULT-LLORCA, F., CORTES, J., GNANT, M., HOUSSAMI, N., POORTMANS, P., RUDDY, K., TSANG, J. & CARDOSO, F. 2019. Breast cancer. Nature Reviews Disease Primers, 5, 66.

HARRIS, L. N., ISMAILA, N., MCSHANE, L. M., ANDRE, F., COLLYAR, D. E., GONZALEZ-ANGULO, A. M., HAMMOND, E. H., KUDERER, N. M., LIU, M. C., MENNEL, R. G., VAN POZNAK, C., BAST, R. C., HAYES, D. F. & AMERICAN SOCIETY OF CLINICAL, O. 2016. Use of Biomarkers to Guide Decisions on Adjuvant Systemic Therapy for Women With Early-Stage Invasive Breast Cancer: American Society of Clinical Oncology Clinical Practice Guideline. Journal of clinical oncology : official journal of the American Society of Clinical Oncology, 34, 1134-1150.

NATIONAL CANCER INSTITUTE SURVEILLANCE EPIDEMIOLOGY AND END RESULTS PROGRAM (SEER). 2014-2018. Cancer Stat Facts: Female Breast Cancer Subtypes [Online]. Available: https://seer.cancer.gov/statfacts/html/breast-subtypes.html [Accessed October 27, 2021].

PEROU, C. M., SØRLIE, T., EISEN, M. B., VAN DE RIJN, M., JEFFREY, S. S., REES, C. A., POLLACK, J. R., ROSS, D. T., JOHNSEN, H., AKSLEN, L. A., FLUGE, Ø., PERGAMENSCHIKOV, A., WILLIAMS, C., ZHU, S. X., LØNNING, P. E., BØRRESEN-DALE, A.-L., BROWN, P. O. & BOTSTEIN, D. 2000. Molecular portraits of human breast tumours. Nature, 406, 747-752.

VIEIRA, A. F. & SCHMITT, F. 2018. An Update on Breast Cancer Multigene Prognostic Tests—Emergent Clinical Biomarkers. 5.
