# Supplementary material for: Leveraging transcriptomics for precision diagnosis: Lessons learned from cancer and sepsis
Source: Front Genet. 2023 Mar 10;14:1100352. doi: 10.3389/fgene.2023.1100352 (PMC10036914; doi:10.3389/fgene.2023.1100352)
Supplement: Supplementary file 2 [file DataSheet1.zip › SupplementaryMaterial_Box2.docx]

Supplementary Material

# Supplementary Box 2: Oncotype DX for breast cancer, a story of success

| - Oncotype DX was developed to be a “fit for purpose” assay. Hence, the test requires small amounts of formalin-fixed and paraffin embedded (FFPE) tissue which are routinely prepared after surgery. - A RT-PCR was developed to measure the expression of 16 cancer-related genes with optimal accuracy in three clinical studies and consistency in probe and primer performances. To address the issue of RNA degradation with storage time and tissue fixation, short and homogeneous in length probes and primers were designed and 5 reference genes were used for normalisation of cancer-gene expression (Cronin et al., 2004). - The 21-gene assay underwent an elaborate analytical validation with high reproducibility across operators, days, instruments and reagents (Cronin et al., 2007). - All procedures are centralised. FFPE samples are shipped to a laboratory in the USA and a report with the RS is sent online within seven to ten calendar days (Oncotype, 2022). - Prognostic utility of the RS was originally validated in a large clinical trial (tamoxifen vs. placebo in ER+/LN- breast tumours) which showed statistically significant (p<0.001) results (Paik et al., 2004). - The predictive ability of the test refers to the addition of adjuvant chemotherapy to reduce the risk of distant recurrence. It has been validated in numerous prospective-retrospective studies and large prospective randomised clinical trials (Nitz et al., 2017, Sparano et al., 2018, Syed, 2020). Several clinical and population-based studies provide good evidence that the estimated RS can be used as a continuous variable to prognosticate the 9-year rate of distant recurrence and predict chemotherapy benefit. - Secondary analyses of the landmark TAILORx, a large (n=9,719) prospective randomised trial, revealed that clinical risk provides additional prognostic information in the group of pre-menopausal women (Sparano et al., 2019). Established clinical factors such as age and tumour features (size and histologic grade) have prognostic information and should be considered for treatment options (Hunter and Longo, 2019, Sparano et al., 2019). - The use of the test has been extended to lymph node positive (LN+ ≤3) cancer (GenomicHealthInc., 2020). - Oncotype DX for predicting benefit from adjuvant chemotherapy in breast cancer is described as a cost-effective method (Rouzier et al., 2013). |
| --- |

# References

CRONIN, M., PHO, M., DUTTA, D., STEPHANS, J. C., SHAK, S., KIEFER, M. C., ESTEBAN, J. M. & BAKER, J. B. 2004. Measurement of gene expression in archival paraffin-embedded tissues: development and performance of a 92-gene reverse transcriptase-polymerase chain reaction assay. Am J Pathol, 164, 35-42.

CRONIN, M., SANGLI, C., LIU, M. L., PHO, M., DUTTA, D., NGUYEN, A., JEONG, J., WU, J., LANGONE, K. C. & WATSON, D. 2007. Analytical validation of the Oncotype DX genomic diagnostic test for recurrence prognosis and therapeutic response prediction in node-negative, estrogen receptor-positive breast cancer. Clin Chem, 53, 1084-91.

GENOMICHEALTHINC. 2020. The majority of postmenopausal HR+, HER2-, N1 patients can be spared chemotherapy when decisions are guided with the Oncotype DX®test [Online]. Available: https://www.oncotypeiq.com/en/announcements/SABCS?gclid=CjwKCAjwjdOIBhA_EiwAHz8xm5ZJqcSfruKbCN1qA_jVGZZusR_zAkL7iUGRj2-GyxtrFhHSR_lSphoCcdAQAvD_BwE [Accessed 12/08/2021].

HUNTER, D. J. & LONGO, D. L. 2019. The Precision of Evidence Needed to Practice "Precision Medicine". N Engl J Med, 380, 2472-2474.

NITZ, U., GLUZ, O., CHRISTGEN, M., KATES, R. E., CLEMENS, M., MALTER, W., NUDING, B., AKTAS, B., KUEMMEL, S., REIMER, T., STEFEK, A., LORENZ-SALEHI, F., KRABISCH, P., JUST, M., AUGUSTIN, D., LIEDTKE, C., CHAO, C., SHAK, S., WUERSTLEIN, R., KREIPE, H. H. & HARBECK, N. 2017. Reducing chemotherapy use in clinically high-risk, genomically low-risk pN0 and pN1 early breast cancer patients: five-year data from the prospective, randomised phase 3 West German Study Group (WSG) PlanB trial. Breast Cancer Res Treat, 165, 573-583.

ONCOTYPE, D. 2022. How to Order the Oncotype DX Breast Recurrence Score® test [Online]. Available: https://www.oncotypeiq.com/en-GB/breast-cancer/healthcare-professionals/oncotype-dx-breast-recurrence-score/how-to-order-a-test [Accessed 08/06/2022].

PAIK, S., SHAK, S., TANG, G., KIM, C., BAKER, J., CRONIN, M., BAEHNER, F. L., WALKER, M. G., WATSON, D., PARK, T., HILLER, W., FISHER, E. R., WICKERHAM, D. L., BRYANT, J. & WOLMARK, N. 2004. A multigene assay to predict recurrence of tamoxifen-treated, node-negative breast cancer. N Engl J Med, 351, 2817-26.

ROUZIER, R., PRONZATO, P., CHÉREAU, E., CARLSON, J., HUNT, B. & VALENTINE, W. J. 2013. Multigene assays and molecular markers in breast cancer: systematic review of health economic analyses. Breast Cancer Res Treat, 139, 621-37.

SPARANO, J. A., GRAY, R. J., RAVDIN, P. M., MAKOWER, D. F., PRITCHARD, K. I., ALBAIN, K. S., HAYES, D. F., GEYER, C. E., DEES, E. C., GOETZ, M. P., OLSON, J. A., LIVELY, T., BADVE, S. S., SAPHNER, T. J., WAGNER, L. I., WHELAN, T. J., ELLIS, M. J., PAIK, S., WOOD, W. C., KEANE, M. M., GOMEZ MORENO, H. L., REDDY, P. S., GOGGINS, T. F., MAYER, I. A., BRUFSKY, A. M., TOPPMEYER, D. L., KAKLAMANI, V. G., BERENBERG, J. L., ABRAMS, J. & SLEDGE, G. W. 2019. Clinical and Genomic Risk to Guide the Use of Adjuvant Therapy for Breast Cancer. 380, 2395-2405.

SYED, Y. Y. 2020. Oncotype DX Breast Recurrence Score(®): A Review of its Use in Early-Stage Breast Cancer. Mol Diagn Ther, 24, 621-632.
