# Supplementary material for: Leveraging transcriptomics for precision diagnosis: Lessons learned from cancer and sepsis
Source: Front Genet. 2023 Mar 10;14:1100352. doi: 10.3389/fgene.2023.1100352 (PMC10036914; doi:10.3389/fgene.2023.1100352)
Supplement: Supplementary file 2 [file DataSheet1.zip › SupplementaryMaterial_Box3.docx]

Supplementary Material

# Supplementary Box 3: Oncotype DX for prostate and colon cancer

| - Oncotype DX has been adapted in prostate and colon cancer to aid treatment decisions (Cucchiara et al., 2018, You et al., 2015). The National Comprehensive Cancer Network (NCCN) guidelines recommend considering Oncotype DX and Decipher (whole-transcriptome assay) in prostate cancer patients with low or favourable intermediate disease and life expectancy greater than or equal to 10 years to inform decisions as to active surveillance or definite therapy (National Comprehensive Cancer Network, 2021a). The evidence from colon cancer studies is currently inadequate to allow the inclusion of multigene assays in guidelines (National Comprehensive Cancer Network, 2021b). - The Oncotype DX Genomic Prostate Score is based on the expression of 12 cancer-genes and 5 reference-genes which also accrued from an original set of 732 candidate genes selected through a meta-analysis of publicly available microarray studies (Klein et al., 2014). - Only small amounts of cancerous tissue are available from prostate needle core biopsies and there is remarkable genetic variability between regions of individual tumours (Knezevic et al., 2013). The challenges are claimed to have been addressed by selecting genes expressed uniformly despite the multifocal nature of the disease and by modifying the PCR protocol to enhance performance with smaller RNA inputs (Klein et al., 2014, Knezevic et al., 2013). - Although data demonstrate strong associations between the Genomic Prostate Score and disease recurrence and grade in patients with localised cancer, the studies have been conducted in subjects with radical prostatectomy within 6 months of diagnosis (Cullen et al., 2015, Klein et al., 2014, Van Den Eeden et al., 2018). Hence, NCCN recognise that the Genomic Prostate Score can contribute additional prognostic information along with conventional clinical parameters such as age, biopsy Gleason score or the Cancer of the Prostate Risk Assessment (CAPRA) score to assist in treatment decisions (National Comprehensive Cancer Network, 2021c). - A validation study designed to investigate the hypothesis that a higher proportion of patients undergoing active surveillance and with low score remain free of clinical progression compared to those with high score would be useful, but difficult to power due to the rarity of this population. Such a study would be able to recommend clinically appreciable cut-off values as recently suggested in patients with intermediate risk cancer (Cullen et al., 2020). |
| --- |

# References

CUCCHIARA, V., COOPERBERG, M. R., DALL'ERA, M., LIN, D. W., MONTORSI, F., SCHALKEN, J. A. & EVANS, C. P. 2018. Genomic Markers in Prostate Cancer Decision Making. Eur Urol, 73, 572-582.

CULLEN, J., KUO, H.-C., SHAN, J., LU, R., ABOUSHWAREB, T. & VAN DEN EEDEN, S. K. 2020. The 17-Gene Genomic Prostate Score Test as a Predictor of Outcomes in Men with Unfavorable Intermediate Risk Prostate Cancer. Urology, 143, 103-111.

CULLEN, J., ROSNER, I. L., BRAND, T. C., ZHANG, N., TSIATIS, A. C., MONCUR, J., ALI, A., CHEN, Y., KNEZEVIC, D., MADDALA, T., LAWRENCE, H. J., FEBBO, P. G., SRIVASTAVA, S., SESTERHENN, I. A. & MCLEOD, D. G. 2015. A Biopsy-based 17-gene Genomic Prostate Score Predicts Recurrence After Radical Prostatectomy and Adverse Surgical Pathology in a Racially Diverse Population of Men with Clinically Low- and Intermediate-risk Prostate Cancer. Eur Urol, 68, 123-31.

KLEIN, E. A., COOPERBERG, M. R., MAGI-GALLUZZI, C., SIMKO, J. P., FALZARANO, S. M., MADDALA, T., CHAN, J. M., LI, J., COWAN, J. E., TSIATIS, A. C., CHERBAVAZ, D. B., PELHAM, R. J., TENGGARA-HUNTER, I., BAEHNER, F. L., KNEZEVIC, D., FEBBO, P. G., SHAK, S., KATTAN, M. W., LEE, M. & CARROLL, P. R. 2014. A 17-gene assay to predict prostate cancer aggressiveness in the context of Gleason grade heterogeneity, tumor multifocality, and biopsy undersampling. Eur Urol, 66, 550-60.

KNEZEVIC, D., GODDARD, A. D., NATRAJ, N., CHERBAVAZ, D. B., CLARK-LANGONE, K. M., SNABLE, J., WATSON, D., FALZARANO, S. M., MAGI-GALLUZZI, C., KLEIN, E. A. & QUALE, C. 2013. Analytical validation of the Oncotype DX prostate cancer assay – a clinical RT-PCR assay optimized for prostate needle biopsies. BMC Genomics, 14, 690.

NATIONAL COMPREHENSIVE CANCER NETWORK. 2021a. NCCN Guidelines Version 2.2021 Prostate Cancer [Online]. Available: https://www.nccn.org/professionals/physician_gls/pdf/prostate.pdf [Accessed 07/08/2021].

NATIONAL COMPREHENSIVE CANCER NETWORK. 2021b. NCCN Guidelines Version 2.2021 Colon Cancer [Online]. Available: https://www.nccn.org/professionals/physician_gls/pdf/colon.pdf [Accessed 07/08/2021].

NATIONAL COMPREHENSIVE CANCER NETWORK. 2021c. Prostate Cancer (Version 1.2022) [Online]. Available: https://www.nccn.org/professionals/physician_gls/pdf/prostate.pdf [Accessed October 21, 2021].

VAN DEN EEDEN, S. K., LU, R., ZHANG, N., QUESENBERRY, C. P., JR., SHAN, J., HAN, J. S., TSIATIS, A. C., LEIMPETER, A. D., LAWRENCE, H. J., FEBBO, P. G. & PRESTI, J. C. 2018. A Biopsy-based 17-gene Genomic Prostate Score as a Predictor of Metastases and Prostate Cancer Death in Surgically Treated Men with Clinically Localized Disease. Eur Urol, 73, 129-138.

YOU, Y. N., RUSTIN, R. B. & SULLIVAN, J. D. 2015. Oncotype DX(®) colon cancer assay for prediction of recurrence risk in patients with stage II and III colon cancer: A review of the evidence. Surg Oncol, 24, 61-6.
